# Optimization of Vortex-Assisted Dispersive Liquid-Liquid Microextraction for the Simultaneous Quantitation of Eleven Non-Anthocyanin Polyphenols in Commercial Blueberry Using the Multi-Objective Response Surface Methodology and Desirability Function Approach

**DOI:** 10.3390/molecules23112921

**Published:** 2018-11-09

**Authors:** Ying Xue, Xian-Shun Xu, Li Yong, Bin Hu, Xing-De Li, Shi-Hong Zhong, Yi Li, Jing Xie, Lin-Sen Qing

**Affiliations:** 1 School of Pharmacy, Collaborative Innovation Center of Sichuan for Elderly Care and Health, Chengdu Medical College, Chengdu 610500, China; xuecher0221@sina.com (Y.X.); saraca1980@126.com (S.-H.Z.); lychengdu@aliyun.com (Y.L.); 2 Chengdu Institute of Biology, Chinese Academy of Sciences, Chengdu 610041, China; li_xingde1975@163.com; 3 Sichuan Provincial Center for Disease Control and Prevention, Chengdu 610041, China; xuxianshun@163.com (X.-S.X.); yongch121@163.com (L.Y.); hubin1258@163.com (B.H.)

**Keywords:** blueberry, non-anthocyanin polyphenol, vortex-assisted dispersive liquid-liquid microextraction, response surface methodology, desirability function approach

## Abstract

In the present study, 11 non-anthocyanin polyphenols, gallic acid, protocatechuate, vanillic acid, syringic acid, ferulic acid, quercetin, catechin, epicatechin, epigallocatechin gallate, gallocatechin gallate and epicatechin gallate—were firstly screened and identified from blueberries using an ultra performance liquid chromatography–time of flight mass spectrography (UPLC-TOF/MS) method. Then, a sample preparation method was developed based on vortex-assisted dispersive liquid-liquid microextraction. The microextraction conditions, including the amount of ethyl acetate, the amount of acetonitrile and the solution pH, were optimized through the multi-objective response surface methodology and desirability function approach. Finally, an ultra performance liquid chromatography–triple quadrupole mass spectrography (UPLC-QqQ/MS) method was developed to determine the 11 non-anthocyanin polyphenols in 25 commercial blueberry samples from Sichuan province and Chongqing city. The results show that this new method with high accuracy, good precision and simple operation characteristics, can be used to determine non-anthocyanin polyphenols in blueberries and is expected to be used in the analysis of other fruits and vegetables.

## 1. Introduction

Blueberry, edible as a fresh fruit, can also be processed into various foods such as blueberry-pizza, blueberry-cake, jam and beverage and so forth. Increasing evidence shows that blueberry and food rich in blueberry have significant benefits to human health [1,2,3,4]. It is generally believed that these benefits derive from rich polyphenols in blueberry [5,6]. At present, the research on blueberry polyphenols is mainly focused on anthocyanins but a few studies have focused on non-anthocyanin polyphenols [7,8,9,10,11], especially in commercial samples. Non-anthocyanin polyphenols are important components in commercial blueberry fruits and contribute to their characteristic flavor. Therefore, in order to fully evaluate the chemical constituents in commercial blueberries, it is necessary to comprehensively study the composition and content of non-anthocyanin polyphenols.

At present, the methods of sample preparation for the determination of polyphenols in food are mainly liquid-liquid extraction (LLE) and solid-phase extraction (SPE) [12,13]. However, LLE and SPE have some inherent disadvantages. For example, LLE requires a large number of toxic solvents and is time-consuming and the SPE materials are expensive. In recent years, the development of dispersive liquid-liquid microextraction (DLLME) can make up the deficiencies of LLE and SPE [14,15]. The principle of DLLME is to form a ternary solvent system using extraction solvent (organic solvent), dispersant (hydrophilic solvent) and sample aqueous solution. The huge contact area allows the emulsification equilibrium and a large number of small droplets to be rapidly formed. Subsequently, the droplets are polymerized into individual organic phases by centrifugation, thereby achieving extraction/enrichment of the target analyte. This method has such advantages as less solvent consumption, simple operation, high enrichment factor and time-saving and so forth. In order to speed up the mass transfer process and shorten the equilibration time, some auxiliary emulsification methods such as vortex-assisted [14], ultrasound-assisted [16], microwave-assisted [17] and air-assisted [18] were also applied to improve the performance of DLLME.

When using the DLLME method for sample preparation, each parameter should be optimized to achieve high-efficient extraction. Inevitably, it is a time-consuming and tedious work to optimize each variable one by one and examine the interaction between variables. Response surface methodology (RSM) can optimize multivariable parameters by reasonable experimental design [19]. Furthermore, combined with the desirability function (DF) approach, RSM could comprehensively optimize multiple parameters to effectively predict the overall best experimental conditions for multiple target analytes simultaneously [20].

In this study, 11 non-anthocyanin polyphenols from blueberry were identified by UPLC-TOF/MS and subsequently determined by UPLC-QqQ/MS using the vortex-assisted dispersive liquid-liquid microextraction (VA-DLLME) method for sample preparation. The optimization based on RSM and DF was performed on VA-DLLME variables including the amount of extraction solvent, the amount of dispersant and pH of the sample solution. Subsequently, UPLC-QqQ/MS was used to determine the content of 11 non-anthocyanin polyphenols in 25 commercial blueberry samples from Sichuan province and Chongqing city. As far as we know, this study is the first assay to describe a non-anthocyanin polyphenols determination method through UPLC-QqQ/MS based on RSM and DF experimental design and optimization, as well as its application in blueberry sample analysis.

## 2. Results and Discussion

### 2.1. Compounds Confirmation by UPLC-TOF/MS

Time-of-flight mass spectrometry (TOF/MS) has the advantages of high-quality accuracy, high sensitivity and high resolution. It can determine the exact mass of the compound, enabling qualitative structure identification in a complex matrix [21,22]. In this study, UPLC-TOF was used to scan polyphenolic compounds in negative ion mode. After referring to the literature on polyphenolic compounds in *Vaccinium* spp. and confirmation by authentic standards under the same chromatographic conditions, 11 non-anthocyanin polyphenols were identified from blueberries as GA (gallic acid, *m*/*z* 169.0143, 0.6 ppm [M − H]^−^), PC (protocatechuate, *m*/*z* 153.0193, 0.1 ppm [M − H]^−^), VA (vanillic acid, *m*/*z* 167.0350, −0.1 ppm [M − H]^−^), SA (syringic acid, *m*/*z* 197.0456, 0.1 ppm [M − H]^−^), FA (ferulic acid, *m*/*z* 193.0505, −0.5 ppm [M − H]^−^), Que (quercetin, *m*/*z* 301.0352, −0.5 ppm [M − H]^−^), C (catechin, *m*/*z* 289.0716, −0.7 ppm [M − H]^−^), EC (epicatechin, *m*/*z* 289.0716, −0.7 ppm [M − H]^−^), ECG (epigallocatechin gallate, *m*/*z* 441.0827, 0 ppm [M − H]^−^), GCG (gallocatechin gallate, *m*/*z* 457.0773, −0.8 ppm [M − H]^−^) and EGCG (epicatechin gallate, *m*/*z* 457.0773, −0.7 ppm [M − H]^−^), respectively.

### 2.2. Optimization of UPLC-QqQ/MS Conditions

**Figure 1:**
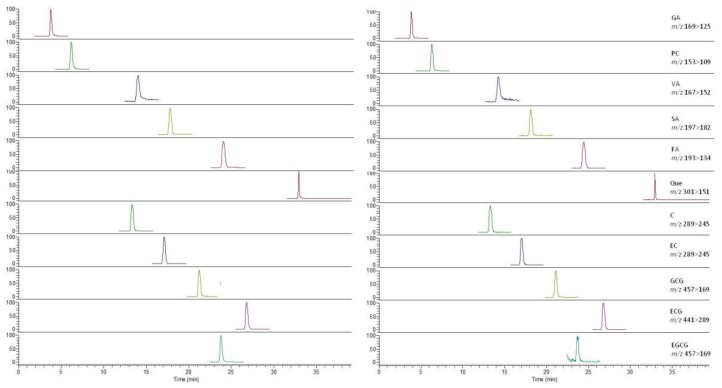
UPLC-MS total ion count chromatograms of 11 non-anthocyanin polyphenols ((**Left**), standard; (**Right**), commercial blueberry sample).

All 11 polyphenols are acidic compounds. Therefore, the acid mobile phase could increase the separating degree, symmetry factor and the number of theoretical plates [23,24]. Considering the ion suppression induced by a high concentration of acid, 0.1% formic acid was finally added into the mobile phase. The performance of 11 polyphenols was compared on three columns of BEH C18 (2.1 mm × 100 mm, 1.7 μm), Shimadzu XR-ODS (2.0 mm × 100 mm, 1.8 μm) and Agilent ZORBAX SB-Aq (3.0 mm × 100 mm, 1.8 μm). The ZORBAX SB-Aq column exhibits a better separation of two pairs of isomers (EC and C, EGCG and ECG) and more symmetrical peak shapes for all of the 11 non-anthocyanin polyphenols can be obtained on it. The MS detection uses negative ion mode for acidic compounds. By optimizing the collision energy (CE) for collision-induced dissociation, two stable product ions with high sensitivity are selected for multiple reaction monitoring (Table S1). The representative mass spectra of blueberry samples are shown in Figure 1.

### 2.3. Optimization of DLLME Condition by Experimental Design

#### 2.3.1. Single Factor Experimental Analysis

Since the extraction conditions have a crucial influence on the extraction results of VA-DLLME, it is essential to define the range of experimental variation and establish corresponding control methods. Therefore, it is necessary to conduct single-factor experiments to determine the variables, including the amount of EtOAc, the amount of ACN and the pH of the solution, which had significant influences on the response. In the present study, the recoveries of 11 non-anthocyanin polyphenols were assessed by means of changing a single variable and fixing two variables. The results are shown in Figure S1. Due to structural differences, the recoveries of the 11 non-anthocyanin polyphenols were different but the optimal values were relatively consistent: when the amount of EtOAc was about 1000 µL, the amount of ACN was about 500 µL and the pH was about 4, there was a high recovery rate. Based on this, the variables and their levels were fixed as shown in Table S2 for the following multi-factor optimization study.

#### 2.3.2. Multi-Response Design and Analysis

Based on the results of single factor experiments, the optimization of VA-DLLME extraction conditions was performed based on Box-Behnken design with three factors and three levels. The experimental design and results are shown in Table 1. Among 11 non-anthocyanin polyphenols, syringic acid (SA) was randomly selected as an example to elucidate the results and the process of analysis. The quadratic regression model of SA was plotted based on response (Y_SA_) versus variables (A, the amount of EtOAc; B, the amount of ACN; and C, the pH of the solution) as below:

Y_SA_ = 94.79 + 15.56A − 5.71B − 7.93C + 3.07AB − 1.95BC − 14.47A^2^ − 10.33B^2^ − 41.16C^2^

The analysis of variance (ANOVA), goodness-of-fit and adequacy of the regression model are summarized in Table 2. The *p*-value in the F-test was used to evaluate the significance level of each variable. The model was considered statistically significant when *p* < 0.05 and was considered highly significant when *p* < 0.01. ANOVA showed that the model’s *F* = 826.42 (*p* < 0.0001), indicating that the experimental error was minimal and the model had highly statistical significance and could be applied to data analysis. Lack of fit *F* = 3.11 (*p* = 0.1511) was not statistically significant, indicating that the experimental error has no significant effect on the model. The results of ANOVA also showed the statistical significance of items A, B and C, interaction items AB and BC and quadratic items A^2^, B^2^ and C^2^ respectively, indicating that the effect of each variable on the response was not only linear but also interactive. The model verified by ANOVA could reflect the relationship between the SA recovery and the amount of EtOAc, the amount of ACN and the pH of the solution. The regression equation could be used to predict and optimize the VA-DLLME process of SA.

**Figure 2:**
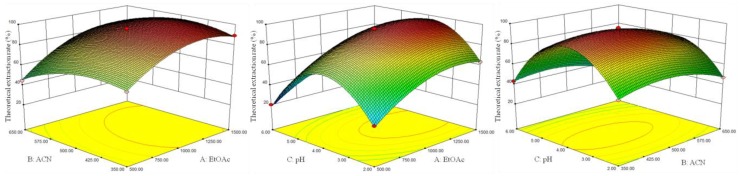
Response surface plots of syringic acid by response (%) versus significant variables (A, the amount of EtOAc; B, the amount of ACN and C, the pH of the solution).

Response surfaces (three-dimensional) were plotted by Design Expert software (8.0.6) to illustrate the interactions of variables for the maximum response. The corresponding 3D response surfaces are shown in Figure 2. Each figure showed the effects of two factors on the SA recovery while the third factor was kept at zero level.

Using the same method, the quadratic regression models of the remaining 10 non-anthocyanin polyphenols were plotted based on response (Y_i_) versus variables (A, the amount of EtOAc; B, the amount of ACN and C, the pH of the solution) as below.

Y_GA_ = 93.70 + 10.26A − 12.82C + 6.86AC − 9.86A^2^ − 6.91B^2^ − 33.64C^2^

Y_PC_ = 91.76 + 9.82A − 13.48C + 2.24BC − 12.92A^2^ − 34.65C^2^

Y_VA_ = 94.65 + 5.13A − 4.58B − 5.81C + 6.52AC − 14.46A^2^ − 2.93B^2^ − 25.52C^2^

Y_FA_ = 93.29 + 7.62A + 2.00B − 12.38C + 3.00AC + 2.25BC − 4.89A^2^ − 3.64B^2^ − 27.89C^2^

Y_Que_ = 88.24 + 7.98A + 1.32B + 4.32AC + 6.64BC − 11.13A^2^ − 7.83B^2^ − 49.88C^2^

Y_C_ = 91.26 + 6.54A − 11.98C + 5.09AC − 1.59BC − 11.83A^2^ − 2.11B^2^ − 25.07C^2^

Y_EC_ = 95.87 + 8.06A − 2.40B − 9.85C + 4.52AC + 1.96BC − 16.80A^2^ − 4.88B^2^ − 28.30C^2^

Y_GCG_ = 93.31 + 7.23A + 1.76B + 10.22C + 4.16BC − 9.71A^2^ − 6.89B^2^ − 50.18C^2^

Y_ECG_ = 92.83 + 7.91A + 1.15B + 10.00C − 2.24AB − 5.43AC + 6.54BC-7.25A^2^ − 8.84B^2^ − 43.04C^2^

Y_EGCG_ = 92.76 + 7.12A − 1.94B + 5.93C + 2.20AB + 1.33AC − 8.85A^2^ − 5.77B^2^ − 41.79C^2^

#### 2.3.3. Optimization Analysis

The desirability function (DF) is a way to optimize multiple responses at the same time. The overall desirability (*D*) was used to search for optimal extraction conditions by the geometric mean of the individual desirability of each response (*d*_i_). The use of the geometric mean ensures that the selected condition could satisfy at least the minimum desired value of all responses and *D* increases as most responses come close to their desired value. For example, if the selected extraction conditions favored some recoveries with high *d* but other recoveries with low *d*, the overall *D* would still be very low. Similarly, the condition having equal but low d, yields a low *D* as well. In this case, the scenario is not desirable. In optimizing the extraction conditions, it will be preferable to maximize the recovery of each polyphenol as high as possible, towards high *D*, so that the selected condition ensures the overall optimal performance.

The higher its sample recovery rate, the better it is; and when multiple components are measured at the same time, the recovery rate of some compounds could be compromised. Therefore, we define *Y_i-_*_max_ in Equation (2) at 100% (theoretical maximum recovery rate) and *Y_i-_*_min_ at 80%. In this way, the *d_i_* value of each compound could be calculated according to Equation (2) and the *D* value of 11 polyphenols could be calculated according to Equation (3), subsequently. With *D* as a new response, its optimal value could be obtained through regression analysis. In the present study, the best conditions for achieving the highest *D* value (0.723) were 1184.52 µL EtOAc, 492.9 µL ACN and solution pH of 3.92. The corresponding theoretical maximum of 11 compounds were GA 96.48%, PC 94.15%, VA 94.88%, SA 99.02%, FA 95.73%, Que 89.44%, C 92.44%, EC 96.93%, GCG 94.03%, ECG 94.34% and EGCG 93.89%, respectively.

#### 2.3.4. Verification of the Predictive Model

In order to verify the applicability of the model equations, six verification experiments (*n* = 6) were performed under optimized conditions. Considering the operability of actual production, the optimal conditions were slightly modified as follows: 1185 µL EtOAc, 493 µL ACN and solution pH of 3.92. Under these conditions, the recoveries of 11 compounds were GA 93.56 ± 1.39%, PC 91.71 ± 1.02%, VA 93.61 ± 1.52%, SA 94.75 ± 0.79%, FA 92.26 ± 1.52%, Que 88.29 ± 1.32%, C 91.13 ± 0.72%, EC 95.76 ± 1.16%, GCG 93.35 ± 0.92% ECG 92.67 ± 1.08% and EGCG 92.71 ± 0.84%. The consistency between the predicted values and experimental values was assessed by Bland-Altman analysis [25]. Using 95% as the confidence interval, the limits of agreement for measurement difference were [−0.0029, 0.0421]. The Bland-Altman plot is depicted in Figure S2, which shows that the experimental values are close to the predicted values. The result confirmed that the models proposed by a combination of RSM and DF methods can be used to optimize the VA-DLLME extraction process.

### 2.4. Analytical Performance of VA-DLLME-UPLC-QqQ/MS

The method validation data are summarized in Table 3, including the calibration curve, linear range, LOD, LOQ, precision, repeatability and recovery. The results indicated that the VA-DLLME-UPLC-QqQ/MS method was satisfactory for the determination of 11 polyphenols in blueberry samples. Besides, the analytical figures of merit were compared with those of several other quantitative methods reported for non-anthocyanin polyphenols determination as shown in Table S3.

### 2.5. Sample Analysis

Twenty-five batches of commercially available blueberry were collected from Sichuan province and Chongqing city. The contents of eleven polyphenols were determined by the present VA-DLLME-UPLC-QqQ/MS method. As shown in Table 4, The difference of GA content was the largest, which was 6.09 times and the difference of VA content was the least, which was 2.46 times. The content of these 11 polyphenols was 208.408–552.326 μg/kg. It was noted that a small amount of EGCG was detected in the four fresh samples picked from local blueberry orchards but samples from shops and supermarkets were not measured. It is estimated that because EGCG is unstable, it decomposes and isomerizes to other catechins during storage [26]. 

## 3. Materials and Methods

### 3.1. Chemical and Apparatus

Eleven authentic reference standards (Figure S3)—gallic acid (GA), protocatechuate (PC), vanillic acid (VA), syringic acid (SA), ferulic acid (FA), quercetin (Que), catechin (C), epicatechin (EC), gallocatechin gallate (GCG), epicatechin gallate (ECG) and epigallocatechin gallate (EGCG)—were purchased from Chengdu Push Bio-technology Co., Ltd. (Chengdu, China). Ultra-pure water was prepared by the Milli-Q water purification system (Millipore, Bedford, MA, USA). HPLC grade acetonitrile (Fisher, Fair Lawn, NJ, USA) was used for UPLC-MS analysis. Other chemicals and reagents of analytical grade were purchased from Chengdu Kelong Chemical Reagent Plant (Chengdu, China).

### 3.2. Preparation of Standard Solution

Eleven stock solutions were prepared by dissolving each authentic reference standard into a 10 mL volumetric flask with methanol, respectively. The exact concentrations were as follows: GA 10.5 mg/mL; PC 9.8 mg/mL; VA 9.9 mg/mL; SA 9.5 mg/mL; FA 9.6 mg/mL; Que 10.4 mg/mL; C 9.6 mg/mL; EC 9.2 mg/ ML; GCG 9.6 mg/mL; EGC 10.8 mg/mL; EGCG 9.8 mg/mL.

The mixed-standard stock solution was prepared by adding 1 mL of each stock solution to a 100 mL volumetric flask and subsequently setting the volume with methanol. Work solutions I-V were obtained by dilution of the mixed-standard stock solution with methanol to the final concentrations of about 1 ng/mL, 2 ng/mL, 5 ng/mL, 10 ng/mL and 20 ng/mL. All the solutions were kept in a refrigerator at 4 °C before use.

### 3.3. Sample Preparation by the DLLME Procedure

Accurately add 1 g blueberry homogenate to a 100 mL volumetric flask; perform ultrasonic extraction with water for 30 min; centrifuge and add 1 mL of supernatant to a 4 mL centrifuge tube; adjust to pH 2–6 using a pH meter by dilute hydrochloric acid; add 500–1500 µL EtOAc and 350–650 µL ACN, then vortex for 30 s. After centrifugation, the upper organic phase was transferred. The extraction was repeated once, combined and the solvent was removed by a Termovap Sample Concentrator. The resulting residue was re-dissolved in 1 mL of methanol and filtered through a 0.45 µm filter for UPLC-MS analysis.

### 3.4. UPLC-MS Analysis

The Shimadzu UPLC system consisted of two pumps (LC-30AD), an auto-sampler (SIL-30AC), a column oven (CTO-30aHE) and an online degasser (DGU-20A^5R^). Chromatographic separation was performed on an Agilent ZORBAX SB-Aq analytical column (3.0 × 100 mm, 1.8 μm) using formic acid solution (0.1%, solvent A) and acetonitrile (solvent B). The gradient elution conditions were as follows: 0~5 min to maintain 5% B and 5~30 min linear increase from 5% B up to 20% B. The column temperature was kept at 40 °C. Two microliters of sample solution was injected into the UPLC system by the auto-sampler.

Qualitative scanning and identification of polyphenols were accomplished with a 4600 Q-TOF mass spectrometer (AB Sciex, Concord, CA, USA) equipped with an electrospray ionization source. Q-TOF/MS analysis was performed in negative mode as follows: ion source gas 1 (GS1) (N_2_) 50 psi, ion source gas 1 (GS1) 50 psi, curtain gas 35 psi, temperature 500 °C, ionspray voltage floating −4500 V. The mass range was set at *m*/*z* 100–1000. The system was operated under Analyst 1.6 and Peak 2.0 (AB Sciex, Concord, CA, USA) and used APCI negative calibration solution to calibrate the instrument′s mass accuracy in real time.

Quantitative analysis of polyphenols was accomplished by a triple quadrupole mass spectrometer (Thermo, TSQ VANTAGE, Waltham, MA USA) equipped with an electrospray ionization source. The MS spectra were acquired in negative ion mode. The quantitative analysis of the target analytes was performed under multi-reaction monitoring (MRM) mode. The mass spectrometry detector (MSD) parameters were as follows: capillary temperature 350 °C, vaporizer temperature 350 °C, sheath gas pressure 40 Arb, aux gas pressure 10 Arb, ion sweep gas pressure 2 Arb, spray voltage −2000 V, discharge current 4.0 µA. Data collection and processing were conducted with Thermo Xcalibur Workstation (Version 2.2, Thermo).

### 3.5. Experimental Design

#### 3.5.1. Single Factor Experimental Design

The most important factors affecting the VA-DLLME assay were the amount of EtOAc, the amount of ACN and the pH of the solution. In the present study, the recoveries of 11 polyphenols were assessed to determine their inflection points by means of changing a single variable and fixing two variables.

#### 3.5.2. Box-Behnken Design

Box-Behnken design (BBD) is a class of (nearly) rotatable second-order design based on three-level incomplete factorial design [27]. On the basis of single-factor experiments, the VA-DLLME conditions were further optimized by BBD of three factors and three levels. Three independent variables—the amount of EtOAc, the mount of ACN and solution pH—were designated as A, B and C, respectively. Furthermore, the range of values was based on the results of the above single factor experiments. The response variables were the recoveries of 11 compounds (Y_1_–Y_11_). There was a total of 17 runs based on BBD design with four center points performed in random order.

### 3.6. Statistical Analysis and Optimization

The parameter and analysis of variance (ANOVA) of the response equations were executed by Design Expert Software (Version 8.0.6). The fitted second-order polynomial model of the response value and independent variables is shown in Equation (1). The significance test of the model was performed and the influence of each parameter on the extraction rates of 11 compounds was analyzed and response surfaces (three-dimensional) were drawn. The statistical significance of variance of each response was tested by ANOVA, where insignificant items were removed from the model and only the data were re-fit to significant parameters (*p* < 0.05) and the surface was constructed. By *p*_lack-of-fit_, the coefficient of determination (R^2^) and their adjusted R^2^ are used to assess the adequacy and quality of the fit.

(1) $$Y_{n}(x)=b_{0}+\sum_{i=1}^{3}b_{i}X_{i}+\sum_{i=1}^{3}b_{ii}X_{i}^{2}+\sum_{i=1}^{2}\sum_{j=i+1}^{3}b_{ij}X_{i}X_{j}\;$$

where *Y_n_* is the predicted response, *b*_0_ is the intercept parameter, *b_i_*, *b_ii and_ b_ij_* are the regression coefficients of linear, quadratic and interaction effects, respectively and *X_i_* and *X_j_* are the independent variables.

The desirability function (DF) is a way to optimize multiple responses at the same time. In this study, DF was used to convert the eleven independent responses into one desirable value (*D*). First of all, each response (recovery value of 11 compounds, *Y_i_*) is firstly converted into a dimensionless desirable value *d_i_* according to Equation (2) and then integrated into *D* by Equation (3). The *D* value increases as the desired response value increases with the range of 0 to 1. *D* = 0 indicates that the response value has seriously deviated from the target value and *D* = 1 indicates that the response value is consistent with the target value. Finally, according to the optimal variable parameter suggested by DF, the experiments with triplicates were conducted and checked to ensure the consistency of the predicted value and the actual value.

(2) $$d_{i}^{}=\{\begin{array}{c} 0\\ \left[\frac{Y_{i}-Y_{i-\min}}{Y_{i-\max}-Y_{i-\min}}\right]\\ 1 \end{array}{}^{r}\begin{array}{l} \;Y_{i}\leq Y_{i-\min}\\ \;Y_{i-\min}<Y_{i}<Y_{i-\max}\\ \;Y_{i}\geq Y_{i-\max} \end{array}\;$$

(3) $$D=\sqrt[{\sum w_{i}}]{d_{1}^{w_{1}}\times d_{2}^{w_{2}}\times d_{3}^{w_{3}}\times d_{i}^{w_{i}}}\;$$

where *di* and *D* are the individual response desirability and overall desirability, respectively; *Y_i_*, *Y_i-_*_min_ and *Y_i-_*_max_ are the actual value of response *i*, the minimum acceptable value for response *i* and the maximum acceptable value for response *i*, respectively; *r* and *w_i_* are the relative weight of the response *i* and both equal 1 in this work.

## 4. Conclusions

In this study, eleven non-anthocyanin polyphenols were firstly screened and identified from blueberries using UPLC-TOF/MS. Then, a vortex-assisted dispersive liquid–liquid microextraction method was developed for sample preparation. With the optimization of the multi-objective response surface methodology and desirability function approach, the best extraction condition was as follows: 1185 µL ethyl acetate, 493 µL acetonitrile and solution pH of 3.92. Finally, the content of 11 polyphenols in 25 batches of blueberry samples from different regions was determined by UPLC-QqQ/MS. This work was designed to be complementary to the work of others and provides a basis for the comprehensive evaluation of active ingredients in blueberries. These results can thus be utilized for extraction standardizations and quality control protocols of blueberry.

## Figures and Tables

**Table 1:** Box-Behnken design matrix and observed responses values of the extraction recovery.

Run	EtOAc/mL	ACN/mL	pH	GA/%	PC/%	VA/%	SA/%	FA/%	Que/%	C/%	EC/%	GCG/%	ECG/%	EGCG/%
1	500	650	4	70.56	66.70	67.73	44.58	77.88	62.34	71.07	62.45	71.01	72.23	66.87
2	1000	650	2	63.73	64.10	68.01	47.23	73.88	25.08	77.61	66.82	23.63	25.57	37.92
3	1000	350	6	38.45	42.72	65.54	43.26	45.13	22.69	53.74	54.63	40.53	43.26	53.66
4	1000	500	4	91.73	93.05	96.68	95.44	91.30	87.13	90.61	97.70	91.98	93.42	93.92
5	1000	350	2	69.13	73.06	76.00	54.35	74.38	35.72	74.52	75.87	29.92	36.35	40.61
6	1000	500	4	92.85	90.09	94.55	93.56	92.40	88.45	92.18	94.57	93.35	92.35	92.89
7	1000	500	4	93.89	91.31	93.12	94.36	94.19	89.80	91.35	94.9	92.69	94.28	91.39
8	1500	350	4	85.00	85.26	87.14	89.24	89.13	75.66	84.24	83.72	80.78	85.73	84.99
9	500	500	6	19.45	17.97	37.20	17.34	37.50	15.05	30.76	27.49	36.20	50.06	39.59
10	1500	500	2	67.24	68.27	59.09	63.46	77.50	30.77	67.8	64.98	28.39	45.88	41.97
11	500	500	2	57.89	48.19	61.86	31.58	68.25	23.44	64.9	58.60	16.47	19.21	30.40
12	500	350	4	65.32	71.02	76.52	62.54	76.38	60.26	70.5	69.12	67.64	65.44	75.16
13	1000	650	6	41.28	42.72	55.22	28.35	53.63	38.61	50.48	53.42	50.88	58.65	48.58
14	1000	500	4	94.12	92.46	93.19	95.89	93.29	86.46	90.49	95.39	94.69	91.24	92.39
15	1500	650	4	86.83	86.54	77.63	83.57	95.63	78.87	83.5	81.46	87.41	83.56	85.51
16	1000	500	4	95.89	91.9	95.69	94.68	95.25	89.35	91.68	96.77	93.84	92.87	93.2
17	1500	500	6	56.24	42.33	60.5	44.25	58.75	39.65	54.02	51.97	52.59	55.01	56.49

**Table 2:** ANOVA for dependent variable: the recovery of SA.

Source	Sum of Squares	df	Mean Square	*F*-Value	*p*-Value	Prob > *F*
Model	11,823.31	9	1313.7	826.42	<0.0001	significant
A-EtOAc	1936.91	1	1936.91	1218.47	<0.0001	
B-ACN	260.6	1	260.6	163.94	<0.0001	
C-pH	502.76	1	502.76	316.28	<0.0001	
AB	37.76	1	37.76	23.75	0.0018	
AC	6.18	1	6.18	3.88	0.0894	
BC	15.17	1	15.17	9.54	0.0176	
A^2^	881.82	1	881.82	554.73	<0.0001	
B^2^	449.45	1	449.45	282.74	<0.0001	
C^2^	7132.12	1	7132.12	4486.67	<0.0001	
Residual	11.13	7	1.59			
Lack of fit	7.79	3	2.6	3.11	0.1511	not significant
Pure error	3.34	4	0.84			
Cor total	11,834.44	16				

**Table 3:** The results of method validation.

Analyte	Regression Equation	Linear Range (ng/mL)	LOD *	LOQ *	Precision (RSD, *n* = 6)		Repeatability (*n* = 6)		Recovery (*n* = 6)	
	(y = ax + b, r^2^)		(ng)	(ng)	Intra-Day (%)	Inter-Day (%)	Mean (μg/kg)	RSD (%)	Mean (%)	RSD (%)
GA	y = 327102x + 293214, 0.9962	1.05–21.0	0.01	0.03	0.59	1.18	9.085	1.27	93.56	1.39
PC	y = 75436x + 19495, 0.9993	0.98–19.6	0.02	0.06	0.96	1.11	10.053	1.34	91.71	1.02
VA	y = 3045x − 254, 0.9998	0.99–19.8	0.3	0.9	2.79	2.92	6.266	1.87	93.61	1.52
SA	y = 3500x − 1177, 0.9997	0.95–19.0	0.1	0.3	1.86	0.95	5.093	0.95	94.75	0.79
FA	y = 67479x − 1274, 0.9999	0.96–19.2	0.1	0.3	1.12	2.95	12.917	0.38	92.26	1.52
Que	y = 442647x − 283610, 0.9986	1.04–20.8	0.01	0.03	1.18	1.88	354.675	0.70	88.29	1.32
C	y = 43132x − 8789, 0.9999	0.96–19.2	0.1	0.3	0.58	1.49	8.295	1.11	91.13	0.72
EC	y = 87001x + 31241, 0.9989	0.92–18.4	0.1	0.3	1.32	1.87	15.177	0.95	95.76	1.16
GCG	y = 49998x − 10512, 0.9997	0.96–19.2	0.1	0.3	1.13	2.12	0.634	2.46	93.35	0.92
ECG	y = 117448x − 15171, 0.9997	1.08–21.6	0.1	0.3	1.36	2.48	9.528	1.88	92.67	1.08
EGCG	Y = 7895x − 1558, 0.9998	0.98–19.6	0.1	0.3	1.04	2.35	0.827	1.31	92.71	0.84

* LOD and LOQ were calculated at the signal-to-noise ratio (S/N) 3 and 10 by a gradual dilution of standard solution, respectively.

**Table 4:** The contents of 11 polyphenols in blueberry samples (μg/kg) (Mean ± SD, *n* = 3).

No	Source	GA	PC	VA	SA	FA	Que	C	EC	GCG	ECG	EGCG	Total
1	orchard, Deyang	9.21 ± 0.15	10.10 ± 0.11	6.38 ± 0.02	5.09 ± 0.10	12.92 ± 0.09	357.54 ± 2.12	8.33 ± 0.11	15.24 ± 0.09	0.66 ± 0.06	9.79 ± 0.07	0.85 ± 0.03	439.096
2	orchard, Chengdu	19.02 ± 0.19	10.87 ± 0.14	7.13 ± 0.06	2.67 ± 0.12	10.09 ± 0.04	332.24 ± 2.38	7.09 ± 0.12	21.12 ± 0.21	1.22 ± 0.09	9.25 ± 0.07	1.72 ± 0.11	422.403
3	orchard, Chengdu	10.55 ± 0.15	4.83 ± 0.10	8.81 ± 0.11	3.25 ± 0.10	6.48 ± 0.11	160.99 ± 1.16	6.48 ± 0.20	37.27 ± 0.20	1.03 ± 0.08	6.44 ± 0.13	1.15 ± 0.05	247.286
4	orchard, Chengdu	18.37 ± 0.18	3.27 ± 0.13	10.112 ± 0.10	2.27 ± 0.14	6.77 ± 0.12	283.86 ± 2.07	8.65 ± 0.20	18.72 ± 0.19	0.88 ± 0.06	11.53 ± 0.10	0.73 ± 0.04	367.165
5	fruit shop, Leshan	13.03 ± 0.16	3.74 ± 0.06	4.39 ± 0.13	5.65 ± 0.10	16.59 ± 0.14	319.60 ± 2.20	8.58 ± 0.12	19.58 ± 0.13	0.85 ± 0.01	13.41 ± 0.09	n.d.	405.423
6	fruit shop, Chengdu	13.92 ± 0.09	5.98 ± 0.10	8.51 ± 0.18	1.86 ± 0.11	6.63 ± 0.08	439.85 ± 2.55	3.77 ± 0.12	36.04 ± 0.06	1.66 ± 0.08	6.14 ± 0.07	n.d.	524.376
7	fruit shop, Chengdu	17.13 ± 0.18	4.74 ± 0.10	10.09 ± 0.15	2.81 ± 0.14	10.50 ± 0.13	390.38 ± 2.61	10.81 ± 0.14	22.16 ± 0.13	1.37 ± 0.08	8.35 ± 0.11	n.d.	478.338
8	fruit shop, Chengdu	19.57 ± 0.13	5.63 ± 0.18	5.71 ± 0.15	3.12 ± 0.12	13.71 ± 0.12	137.62 ± 1.84	9.08 ± 0.08	13.56 ± 0.110	0.67 ± 0.05	8.63 ± 0.13	n.d.	217.294
9	fruit shop, Yibing	12.24 ± 0.21	5.72 ± 0.15	8.21 ± 0.13	4.69 ± 0.09	15.30 ± 0.15	430.71 ± 2.63	3.95 ± 0.11	15.26 ± 0.12	0.57 ± 0.05	9.22 ± 0.10	n.d.	505.877
10	fruit shop, Deyang	11.02 ± 0.11	4.77 ± 0.16	8.74 ± 0.15	5.83 ± 0.12	16.40 ± 0.10	231.83 ± 2.07	9.23 ± 0.14	26.41 ± 0.11	1.56 ± 0.07	6.54 ± 0.12	n.d.	322.336
11	fruit shop, Suining	13.46 ± 0.12	5.44 ± 0.09	5.67 ± 0.12	1.89 ± 0.11	15.70 ± 0.09	220.23 ± 2.59	8.89 ± 0.10	17.34 ± 0.10	1.28 ± 0.08	13.01 ± 0.10	n.d.	302.876
12	fruit shop, Neijiang	14.41 ± 0.16	8.22 ± 0.10	5.80 ± 0.18	3.51 ± 0.12	8.14 ± 0.07	134.57 ± 1.67	6.30 ± 0.10	21.69 ± 0.15	1.54 ± 0.06	4.22 ± 0.10	n.d.	208.408
13	fruit shop, Mianyang	11.91 ± 0.08	8.98 ± 0.16	7.85 ± 0.15	2.35 ± 0.09	14.45 ± 0.134	394.96 ± 2.56	7.41 ± 0.15	19.89 ± 0.08	0.80 ± 0 .03	12.83 ± 0.13	n.d.	481.433
14	fruit shop, Nanchong	19.52 ± 0.12	9.97 ± 0.11	8.65 ± 0.18	3.73 ± 0.10	10.23 ± 0.10	376.28 ± 2.00	11.15 ± 0.08	33.17 ± 0.10	0.91 ± 0.05	9.86 ± 0.14	n.d.	483.473
15	fruit shop, Langzhong	7.80 ± 0.14	5.58 ± 0.07	7.67 ± 0.13	4.68 ± 0.08	14.08 ± 0.11	180.87 ± 1.54	8.71 ± 0.10	28.82 ± 0.14	1.10 ± 0.08	6.03 ± 0.04	n.d.	265.320
16	fruit shop, Chongqing	14.97 ± 0.12	7.88 ± 0.13	6.02 ± 0.15	4.69 ± 0.10	5.89 ± 0.10	357.77 ± 2.23	4.32 ± 0.12	23.25 ± 0.11	1.06 ± 0.07	9.86 ± 0.11	n.d.	435.707
17	fruit shop, Chongqing	18.02 ± 0.08	6.91 ± 0.10	10.26 ± 0.10	3.06 ± 0.11	13.93 ± 0.09	229.77 ± 1.84	8.35 ± 0.11	16.92 ± 0.10	1.27 ± 0.05	4.02 ± 0.09	n.d.	312.492
18	supermarket, Deyang	3.25 ± 0.17	9.25 ± 0.12	8.86 ± 0.09	2.71 ± 0.13	12.60 ± 0.13	302.55 ± 1.83	10.30 ± 0.18	23.5 7 ± 0.14	0.80 ± 0.04	13.03 ± 0.07	n.d.	386.919
19	supermarket, Chengdu	19.82 ± 0.11	7.12 ± 0.09	8.21 ± 0.18	4.59 ± 0.08	15.74 ± 0.14	450.23 ± 3.27	5.74 ± 0.90	28.95 ± 0.14	1.16 ± 0.07	10.76 ± 0.09	n.d.	552.326
20	supermarket, Chengdu	14.35 ± 0.12	5.98 ± 0.09	8.27 ± 0.10	3.87 ± 0.14	10.19 ± 0.08	397.70 ± 2.14	8.61 ± 0.11	18.29 ± 0.06	1.07 ± 0.07	13.03 ± 0.13	n.d.	481.339
21	supermarket, Chengdu	16.68 ± 0.22	8.77 ± 0.16	7.69 ± 0.10	5.16 ± 0.06	8.50 ± 0.10	405.34 ± 1.85	4.81 ± 0.10	28.00 ± 0.12	1.61 ± 0.02	4.88 ± 0.13	n.d.	491.432
22	supermarket, Chengdu	12.95 ± 0.18	7.37 ± 0.12	4.17 ± 0.07	2.75 ± 0.13	7.12 ± 0.09	387.03 ± 2.31	6.55 ± 0.10	18.13 ± 0.11	0.73 ± 0.03	7.97 ± 0.10	n.d.	454.761
23	supermarket, Mianyang	7.66 ± 0.12	5.51 ± 0.09	7.06 ± 0.14	3.70 ± 0.08	10.80 ± 0.11	261.42 ± 2.10	10.34 ± 0.11	20.37 ± 0.12	0.88 ± 0.05	11.31 ± 0.11	n.d.	339.054
24	supermarket, Chongqing	11.96 ± 0.16	7.16 ± 0.12	10.11 ± 0.11	4.70 ± 0.16	7.54 ± 0.11	223.72 ± 1.74	4.29 ± 0.11	32.41 ± 0.13	1.07 ± 0.07	11.52 ± 0.11	n.d.	314.477
25	supermarket, Chongqing	13.92 ± 0.16	7.26 ± 0.15	8.09 ± 0.05	4.19 ± 0.19	11.51 ± 0.10	319.40 ± 2.21	8.06 ± 0.09	25.40 ± 0.11	1.14 ± 0.04	9.42 ± 0.12	n.d.	408.386

## Supplementary Materials

The following are available online.
