# Supplementary material for: Optimization of Vortex-Assisted Dispersive Liquid-Liquid Microextraction for the Simultaneous Quantitation of Eleven Non-Anthocyanin Polyphenols in Commercial Blueberry Using the Multi-Objective Response Surface Methodology and Desirability Function Approach
Source: Molecules. 2018 Nov 9;23(11):2921. doi: 10.3390/molecules23112921 (PMC6278316; doi:10.3390/molecules23112921)
Supplement: Supplementary file 1 [file molecules-23-02921-s001.pdf]

Supplementary material

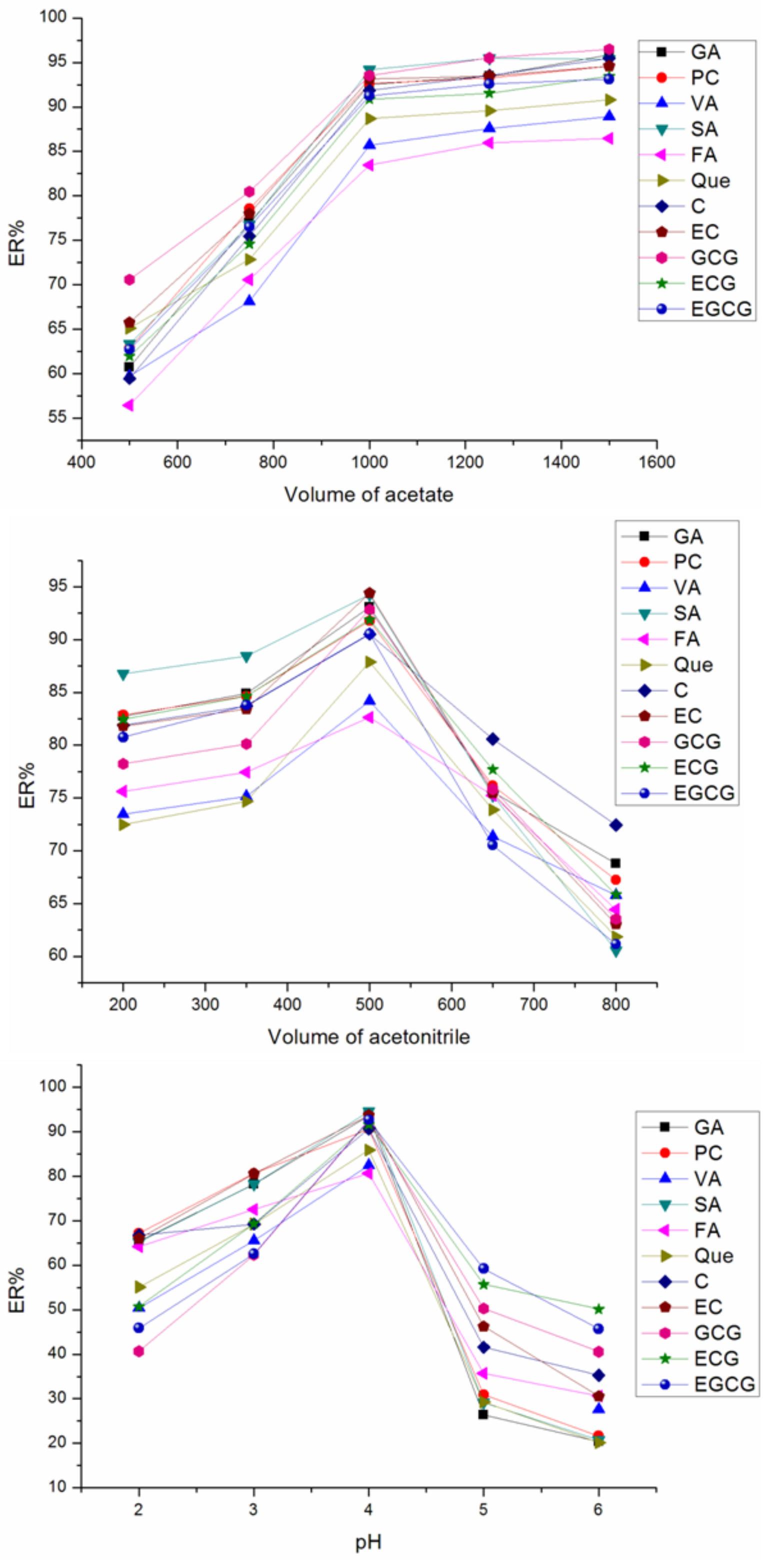

Figure S1 The extraction rate (%) of 11 polyphenols assessed by single factor experimental analysis (the fixed values of parameters from top to bottom were: ACN 500 μL, pH 4; EtOAc 1000 μL, pH 4; and ACN 500 μL, EtOAc 1000 μL, respectively.

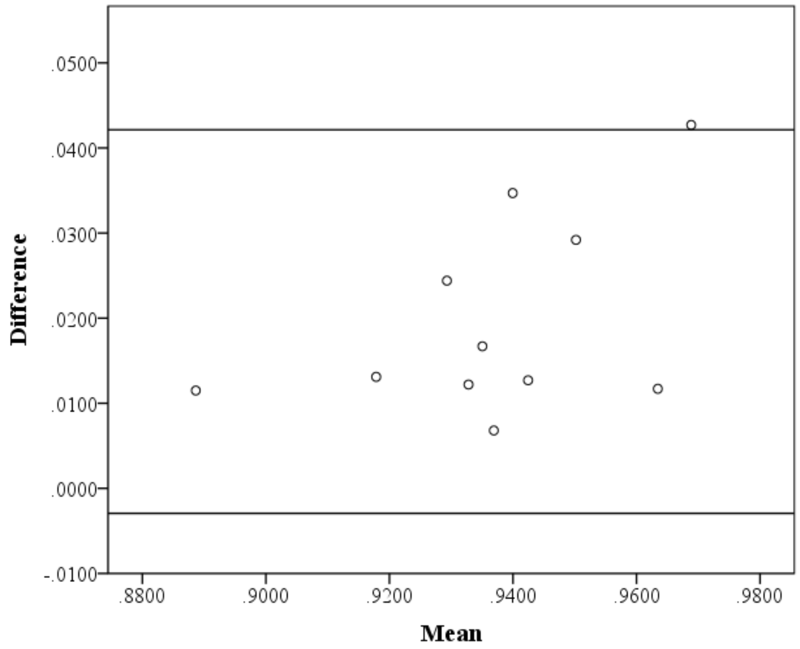

Figure S2 Bland-Altman plot of predicted values and experimental values.

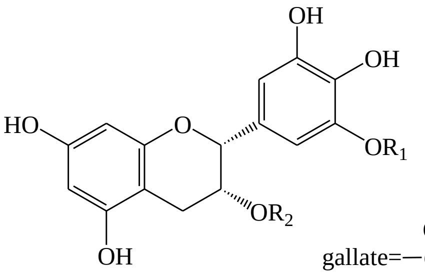

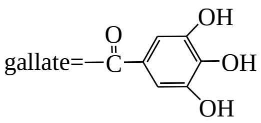

Epicatechin (EC): R_1=R_2=H
Epicatechin gallate (ECG): R_1=H, R_2=gallate
Epigallocatechin gallate (EGCG): R_1=OH, R_2=gallate

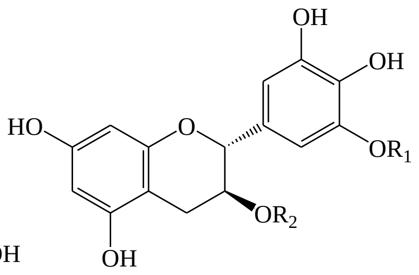

Catechin (C): R_1=R_2=H
Gallocatechin gallate (GCG): R_1=OH, R_2=gallate

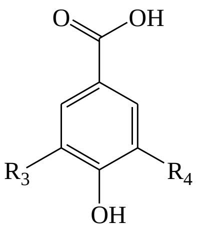

Gallic acid (GA): R_3=R_4=OH
Protocatechuate (PC): R_3=H, R_4=OH
Vanillic acid (VA): R_3=H, R_4=OCH_3
Syringic acid (SA): R_3=R_4=OCH_3

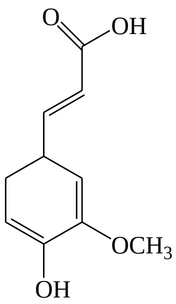

Ferulic acid (FA)

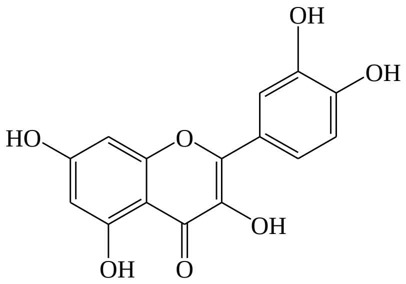

Quercetin (Que)

Figure S3 The chemical structures of eleven polyphenols.

Table S1 MS/MS parameters for the analysis of 11 polyphenols in LC-QqQ/MS analysis.

Compound	Precursor ion (*m*/*z*)	Product ion (*m*/*z*)	CE (V)	S-Lens
GA	169	81#	20	67
		125*	18	67
PC	153	91#	28	71
		109*	17	71
VA	167	108#	20	65
		152*	16	65
SA	197	121#	19	82
		182*	16	82
FA	193	134*	17	68
		178#	15	68
Que	301	107#	30	93
		151*	23	93
C	289	203#	22	113
		245*	17	113
EC	289	123#	33	104
		245*	17	104
GCG	457	125#	43	129
		169*	19	129
ECG	441	169#	24	123
		289*	20	123
EGCG	457	125#	43	138
		169*	20	138

note: *quantitative ion, #qualitative ion

Table S2 Independent variables and their levels in Box-Behnken design.

Independent variables	Symbol	Level		
		-1	0	1
Amount of EtOAc (μL)	A	500	1000	1500
Amount of ACN (μL)	B	350	500	650
Solution pH	C	2	4	6

Table S3 Comparison of analytical methods reported for determination of bovine serum albumin

	liquid-liquid extraction, HPLC-MS analysis [Ref S1]			solid-phase extraction, HPLC-MS analysis [Ref S2]			extracted by ethanol for 12 h, HPLC-DAD analysis [Ref S3]			vortex-assisted dispersive liquid-liquid microextraction, HPLC-MS analysis [This work]		
	Recovery	LOD	LOQ	Recovery	LOD	LOQ	Recovery	LOD	LOQ	Recovery	LOD	LOQ
GA	-*	0.25 pmol	-	-	3 ng/ml	10 ng/ml	-	-	-	93.56%	0.01 ng	0.03 ng
PC	-	-	-	-	-	-	-	-	-	91.71%	0.02 ng	0.06 ng
VA	-	-	-	-	100 ng/ml	400 ng/ml	-	-	-	93.61%	0.3 ng	0.9 ng
SA	-	0.5 pmol	-	-	25 ng/ml	60 ng/ml	-	-	-	94.75%	0.1 ng	0.3 ng
FA	-	0.5 pmol	-	-	60 ng/ml	200 ng/ml	-	-	-	92.66%	0.1 ng	0.3 ng
Que	-	-	-	-	-	-	-	-	-	88.29%	0.01 ng	0.03 ng
C	-	-	-	-	-	-	-	2.73 μg/mL	9.11 μg/mL	91.13%	0.1 ng	0.3 ng
EC	-	-	-	-	-	-	91%	1.77 μg/mL	5.91 μg/mL	95.76%	0.1 ng	0.3 ng
GCG	-	-	-	-	-	-	-	-	-	93.35%	0.1 ng	0.3 ng
ECG	-	-	-	-	-	-	-	-	-	92.67%	0.1 ng	0.3 ng
EGCG	-	-	-	-	-	-	-	-	-	92.71%	0.1 ng	0.3 ng

*unreported

[Ref S1] Ayaz, F.A., et al., Separation, characterization, and quantitation of phenolic acids in a little-known blueberry (Vaccinium arctostaphylos L.) fruit by HPLC-MS. Journal of Agricultural and Food Chemistry, 2005. 53(21): 8116-8122.

[Ref S2] Su, X., et al., Phenolic acid profiling, antioxidant, and anti-inflammatory activities, and miRNA regulation in the polyphenols of 16 blueberry samples from China. Molecules, 2017. 22(2).

[Ref S3] Harris, C.S., et al., A single HPLC-PAD-APCI/MS method for the quantitative comparison of phenolic compounds found in leaf, stem, root and fruit extracts of Vaccinium angustifolium. Phytochemical Analysis, 2007. 18(2): 161-169.
